# A late fusion multi-task learning for respiratory waveform and rate estimation from photoplethysmography

**DOI:** 10.1371/journal.pone.0353203

**Published:** 2026-07-10

**Authors:** Minh Nhut Ho, Kien Trong Nguyen

**Affiliations:** Posts and Telecommunications Institute of Technology, Ho Chi Minh City, Vietnam; Coventry University, UNITED KINGDOM OF GREAT BRITAIN AND NORTHERN IRELAND

## Abstract

Continuous respiratory monitoring enables early detection of physiological deterioration, yet conventional capnography remains impractical for prolonged use. Photoplethysmography (PPG) offers a non-invasive alternative that encodes respiratory information through baseline wander (respiratory-induced intensity variation; RIIV), amplitude modulation (respiratory-induced amplitude variation; RIAV), and frequency modulation (respiratory-induced frequency variation; RIFV) of the pulsatile waveform. Existing PPG-based deep learning approaches, whether operating on the raw signal or on these physiological modulations, are limited to single-task architectures that estimate either respiratory rate or reconstruct the respiratory waveform in isolation, without jointly addressing both outputs. We propose a late fusion multi-task framework in which dedicated encoder branches independently process each modulation before fusion, and dual decoders simultaneously reconstruct the respiratory waveform and estimate the respiratory rate. The framework was evaluated on the CapnoBase (*n* = 42) and BIDMC (*n* = 52) benchmarks across multiple training strategies. For respiratory-rate estimation, the best transfer-learning configurations achieved a mean absolute error (MAE) of 2.27 bpm on CapnoBase and 1.33 bpm on BIDMC. For waveform reconstruction, the corresponding MAE values were 19.00% and 20.90%, with moderate correlations (*r* = 0.662 and *r* = 0.591, respectively). Sequential transfer learning consistently outperformed all other strategies, whereas pooled training degraded both outputs, demonstrating that capnography-derived and impedance-derived waveforms are not interchangeable training targets. These findings establish that short-window PPG can simultaneously support respiratory-rate estimation and waveform reconstruction, when reference signal compatibility is explicitly addressed in multi-task training.

## Introduction

Continuous respiratory monitoring, encompassing both the tracking of breathing rate and the reconstruction of respiratory waveform morphology, is essential for early disease detection and patient management [1]. Changes in respiratory patterns often serve as the earliest indicator of clinical deterioration, preceding adverse events such as cardiac arrest and in-hospital mortality [2]. This recognition has led to the integration of respiratory parameters into early warning scoring systems such as the National Early Warning Score (NEWS) for assessing disease severity in emergency settings [3]. Yet in routine clinical practice, respiration remains the most poorly recorded vital sign [4], and conventional measurement methods (including capnography and impedance pneumography) are cumbersome and ill-suited for long-term use [5]. The advent of wearable sensor technology has increased interest in automated alternatives. Recent propensity-matched analyses confirm that continuous monitoring on general wards reduces unplanned ICU transfers and improves patient outcomes [6], with similar benefits observed when comparing continuous versus periodic monitoring for deterioration detection [7]. These findings highlight a clear need for unobtrusive, non-invasive solutions capable of providing both respiratory rate estimates and waveform-level information in real time.

Photoplethysmography (PPG) provides a promising approach toward this goal. Originally developed for pulse oximetry and heart rate measurement [8], PPG has since been recognized as a versatile platform for extracting a broader range of physiological parameters [9]. Its low cost, optical simplicity, and seamless integration into consumer wearables make it well-suited for ambulatory respiratory monitoring [10]. However, PPG signals are susceptible to motion artifacts, ambient light changes, and poor skin contact [11], all of which can degrade the quality of derived respiratory estimates. Signal quality assessment [12] and artifact removal techniques [13] have therefore become important preprocessing steps. Despite these challenges, multiple studies have confirmed that PPG waveforms carry respiratory information through well-characterized physiological modulation mechanisms [14].

These mechanisms arise from the coupling between respiratory and cardiovascular systems [15,16]. Three main modulation pathways have been identified and their time-frequency signatures characterized [17]: respiratory-induced intensity variation (RIIV, also termed baseline wander, BW) [18,19], respiratory-induced amplitude variation (RIAV, also termed amplitude modulation, AM) [20], and respiratory-induced frequency variation (RIFV, also termed frequency modulation, FM) [21,22]. The physiological mechanisms underlying each modulation are described in detail in the Methods section. Together, these modulations form the physiological basis for extracting both respiratory waveforms and rate from PPG signals.

Building on this physiological foundation, numerous traditional signal processing methods have been developed for PPG-based RR estimation. Dehkordi et al. showed that instantaneous RR can be reliably extracted by combining estimates from multiple respiratory-induced variations [23]. Bayesian tracking of intrinsic modes in time-frequency spectra has provided robust frequency estimates under non-stationary conditions [24]. Further investigations have revealed that extraction accuracy depends on sensor placement site [25] and body position [26]. In terms of signal decomposition, ensemble empirical mode decomposition (EEMD) combined with principal component analysis has been applied to separate respiratory and cardiac components [27], while complementary EEMD with independent component analysis and non-negative matrix factorization has extended this approach [28]. Kalman filtering integrated with EEMD has removed the reliance on fiducial point detection [29], and wavelet-based algorithms have enabled implementation on resource-constrained embedded platforms [30]. More recently, harmonic analysis with sequential temporal fusion has achieved high accuracy for RR estimation from PPG baseline wandering [31]. Fusion strategies that combine quality-weighted estimates from multiple modulations have shown consistent improvements over single-modulation methods [32], building on earlier data fusion frameworks [33]. Adaptive lattice notch filters have also enabled real-time RR tracking from PPG [34]. These classical methods established a solid understanding of how respiratory information is encoded in PPG and how it can be reliably recovered.

The transition from hand-crafted features to learned representations has yielded substantial improvements. For respiratory rate estimation, Bian et al. proposed an end-to-end ResNet architecture that achieved a mean absolute error (MAE) of 2.5 ± 0.6 breaths per minute (bpm) on benchmark datasets [35]. Rather than processing raw PPG, Lampier et al. explicitly extracted BW, AM, and FM modulations and used them as structured inputs to a deep neural network [36]. Lightweight architectures suitable for embedded deployment have also been investigated [37], alongside evaluations of LSTM and other sequence models for respiratory rate prediction [38]. For respiratory waveform reconstruction, Davies and Mandic developed a convolutional corr-encoder that recovers full respiratory waveforms directly from PPG [39]. Shuzan et al. proposed PPG2RespNet, a U-Net++-inspired model that achieved Pearson correlations of 0.94–0.96 with reference respiratory signals across three public datasets [40]. Multiscale residual CNNs such as RRWaveNet have shown robust cross-dataset generalization through transfer learning [41]. Patient-independent estimation has been achieved using spectro-temporal features derived solely from PPG without discarding any signal windows [42]. Roy et al. combined deep autoencoders with optimal IMF weighting for both waveform reconstruction and rate tracking [43], while Chin et al. developed a CNN-LSTM model that operates on input windows as short as 7 seconds [44]. Beyond conventional neural networks, spiking neural networks have been explored for energy-efficient edge deployment [45]. Hybrid SqueezeNet-Transformer architectures with multi-scale attention have been proposed for FPGA-based real-time inference [46], and Transformer-based models have been applied to capture long-range temporal dependencies in respiratory signals [47].

Despite these advances, nearly all existing methods employ single-task architectures that estimate either respiratory rate or reconstruct the respiratory waveform in isolation. Multi-task learning, which simultaneously optimizes multiple related objectives through shared representations, has shown promise in related physiological signal processing tasks, including joint PPG-ECG respiratory estimation [48], blood pressure estimation from physiological signals [49], and most recently, simultaneous heart rate and respiratory rate estimation from PPG [50]. However, the application of multi-task learning to joint respiratory waveform reconstruction and rate estimation, with explicit use of physiologically grounded BW, AM, and FM features, has not yet been explored. This gap is significant because waveform reconstruction and rate estimation represent complementary views of the same underlying physiological process: accurate waveform recovery inherently captures the temporal structure from which rate can be derived, while rate-level supervision can regularize waveform predictions toward physiologically plausible breathing patterns.

This study addresses these gaps through a unified pipeline that integrates physiologically informed feature extraction, modulation-specific encoding, and multi-task decoding. At the input stage, we adopt RIIV, RIAV, and RIFV modulations as structured network inputs, extending the physiologically informed design of Lampier et al. [36] from single-task rate estimation to a multi-task context in which feature interpretability is maintained from input through to both output tasks. At the encoding stage, a late fusion architecture processes each modulation through a dedicated encoder branch before integration, preserving modulation-specific representations that a monolithic architecture would conflate. At the decoding stage, dual decoder branches, inspired by the encoder-decoder paradigm of Davies and Mandic [39] for respiratory waveform recovery, jointly optimize waveform reconstruction and rate estimation through independent loss terms. The two tasks share encoder representations but are decoded independently, allowing each branch to specialize: the waveform decoder learns to recover temporal morphology, while the rate decoder extracts periodicity information from the same shared features. This multi-task formulation encourages the encoder to learn richer respiratory representations than either task alone would require. In addition to evaluating the proposed architecture, we compare five training strategies and show that pooling data from datasets with different ground truth modalities (capnography versus impedance pneumography) degrades performance due to conflicting waveform reconstruction targets, whereas sequential transfer learning between domains consistently yields the best results.

## Materials and methods

### Datasets

**Fig 1:**
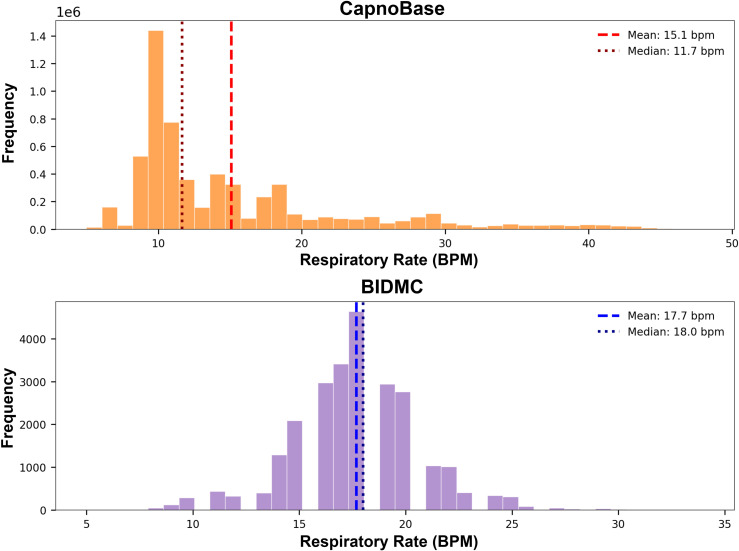
Respiratory rate distribution in the CapnoBase and BIDMC datasets. Upper panel: CapnoBase dataset showing a positively skewed distribution (mean: 15.1 bpm, median: 11.7 bpm) reflecting the pediatric population. Lower panel: BIDMC dataset showing a narrower, more symmetric distribution (mean: 17.7 bpm, median: 18.0 bpm) characteristic of adult intensive care patients.

We evaluated the proposed framework using two publicly available benchmark datasets that employ different ground truth modalities, capnography versus impedance pneumography, enabling investigation of cross-modality generalization. Fig 1 illustrates the respiratory rate distributions for both datasets.

The CapnoBase dataset [16,51] comprises 42 subjects (29 children, 13 adults) recorded during elective surgery. Each 8-minute recording includes simultaneous PPG and capnography signals sampled at 300 Hz. Respiratory rates span 5–48 breaths per minute (mean: 15.1 bpm, median: 11.7 bpm), with the wide range and positive skew reflecting the pediatric population. This distribution poses challenges for algorithms, as PPG signals attenuate respiratory information at higher breathing rates. All data were retained without artifact exclusion to enable realistic performance assessment.

The BIDMC dataset [32,52] contains 53 adult intensive care patients from the MIMIC-II database. Each 8-minute recording includes PPG and impedance pneumography signals sampled at 125 Hz. Respiratory rates range from 5 to 34 breaths per minute (mean: 17.7 bpm, median: 18.0 bpm), with a narrower, more symmetric distribution compared to CapnoBase. Performance assessment is complicated by arterial blood pressure oscillations at 0.1 Hz that overlap with respiratory frequencies. One subject (bidmc_13) was excluded due to >75% missing annotations, yielding 52 subjects for analysis [47].

The two ground truth modalities exhibit distinct waveform characteristics that are central to this study’s findings. Capnography measures expired CO_2_, producing sharp waveform transitions at breath boundaries with distinct plateau phases. Impedance pneumography measures thoracic impedance, producing smoother sinusoidal waveforms that track chest wall movement. These morphological differences directly affect multi-task waveform reconstruction objectives, as discussed in the Results and Discussion sections.

### Signal preprocessing

Three physiological mechanisms couple respiration to the PPG waveform [15,16]. Baseline wander (BW), or respiratory-induced intensity variation (RIIV), reflects respiratory modulation of venous return and tissue blood volume: during inspiration, decreasing intrathoracic pressure increases venous return to the right heart while reducing peripheral venous volume, producing slow baseline oscillations at the respiratory frequency [18]. The influence of different ventilation modes on this component has been characterized under controlled conditions [19]. Amplitude modulation (AM), or respiratory-induced amplitude variation (RIAV), originates from cyclic variations in stroke volume across the breathing cycle, which modulate pulse-wave amplitude [20]. Frequency modulation (FM), or respiratory-induced frequency variation (RIFV), manifests as respiratory sinus arrhythmia, a beat-to-beat heart rate fluctuation driven by parasympathetic modulation of cardiac pacing [21,22] and thought to facilitate pulmonary gas exchange. All three modulations operate within the respiratory frequency band (0.1 to 0.6 Hz) and are therefore separable from cardiac components (0.8 to 3.0 Hz) through bandpass filtering and dedicated extraction algorithms described below.

**Fig 2:**
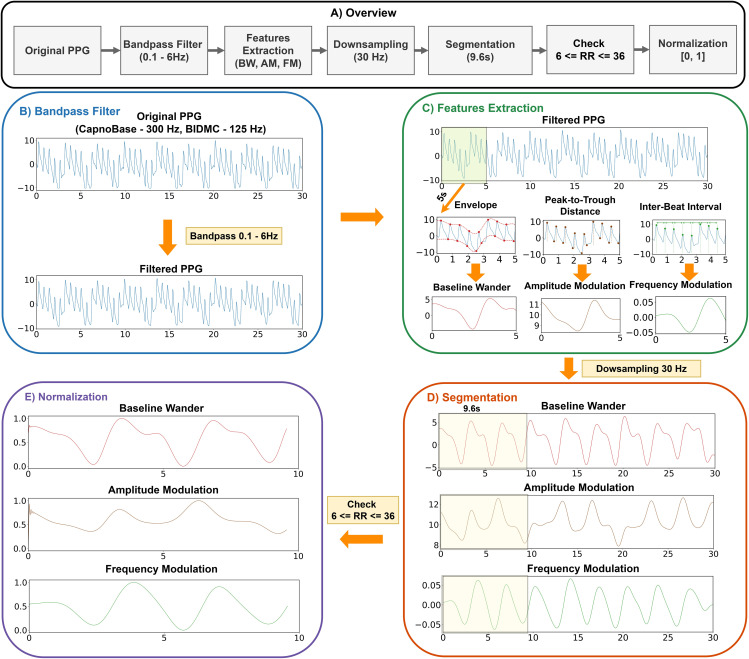
Signal preprocessing workflow for PPG feature extraction. A: Overview of the seven-stage pipeline from raw PPG to normalized feature channels. B: Bandpass filtering (0.1 to 6.0 Hz) of the original PPG signal. C: Extraction of three respiratory modulations, specifically baseline wander, amplitude modulation, and frequency modulation, from the filtered PPG via envelope detection, peak-to-trough distance measurement, and interbeat interval analysis, respectively. D: Segmentation into 9.6-second windows. E: Per-segment min-max normalization to the [0,1] range.

The preprocessing pipeline extracts these three physiologically grounded modulations from the raw PPG signal. Fig 2 illustrates the complete workflow comprising seven sequential stages from raw PPG acquisition through feature extraction, downsampling, segmentation, quality control, and normalization.

*Signal filtering.* The raw PPG is bandpass-filtered between 0.1 and 6.0 Hz using a second-order Butterworth applied bidirectionally for zero phase distortion, which gives an effective fourth-order amplitude response across the cardiac and respiratory bands. The RIAV channel is additionally low-pass filtered at 0.5 Hz and the RIFV channel is bandpass-filtered at 0.1 to 0.6 Hz with the same second-order Butterworth specification.

*Modulation extraction.* Three respiratory modulation channels are derived from the peak-trough series detected on the filtered PPG by adaptive amplitude thresholding: RIIV from the cubic-spline interpolated envelope mean, RIAV from the peak-to-trough amplitude series subsequently low-pass filtered at 0.5 Hz, and RIFV from cubic-spline interpolation of interbeat intervals subsequently bandpass-filtered at 0.1 to 0.6 Hz.

*Segmentation and quality control.* The four channels (filtered PPG, RIIV, RIAV, RIFV) are polyphase-decimated to 30 Hz and segmented into non-overlapping 9.6-second windows of 288 samples. Segments whose mean respiratory rate falls outside the physiological 6–36 bpm range are excluded.

**Fig 3:**
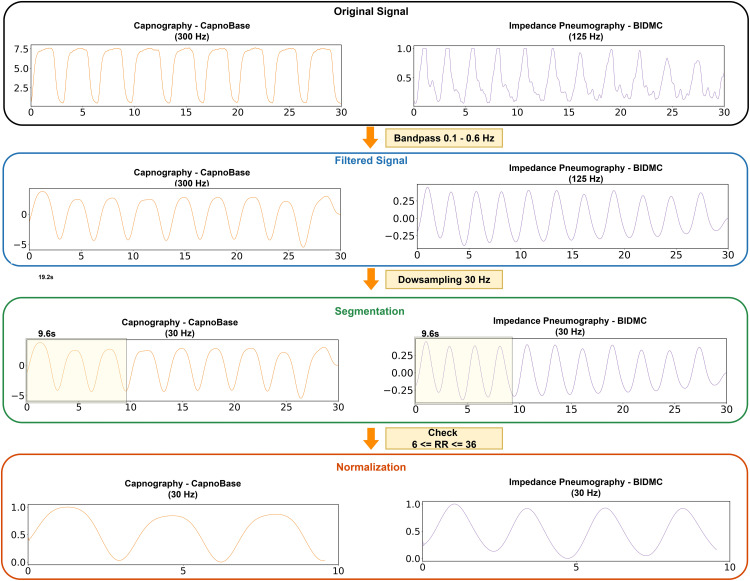
Ground truth signal preprocessing workflow for CapnoBase and BIDMC datasets. Left column: capnography signals (CapnoBase) showing sharp breath-boundary transitions. Right column: impedance pneumography signals (BIDMC) showing smoother sinusoidal patterns. Processing stages from top to bottom: original signal, bandpass-filtered signal (0.1 to 0.6 Hz), downsampled to 30 Hz, segmented into 9.6-second windows, and normalized to [0,1].

*Ground truth preprocessing.* Ground truth signals received separate preprocessing to ensure compatibility with extracted PPG features. Fig 3 illustrates the parallel processing pipelines for capnography (CapnoBase) and impedance pneumography (BIDMC). We evaluated two preprocessing conditions: unfiltered ground truth retaining original frequency content including harmonics, and bandpass-filtered ground truth (0.1 to 0.6 Hz) isolating the primary respiratory band to match the frequency range of extracted PPG features.

*Normalization.* Feature channels and ground-truth waveforms are min-max normalized to [0,1] per segment, preserving relative temporal dynamics within each segment while ensuring consistent input scales across diverse patients. Respiratory rate values underwent global normalization using dataset-wide bounds (6–36 bpm) to maintain consistent regression target scaling across all training samples [47].

### Proposed multi-task architecture

**Fig 4:**
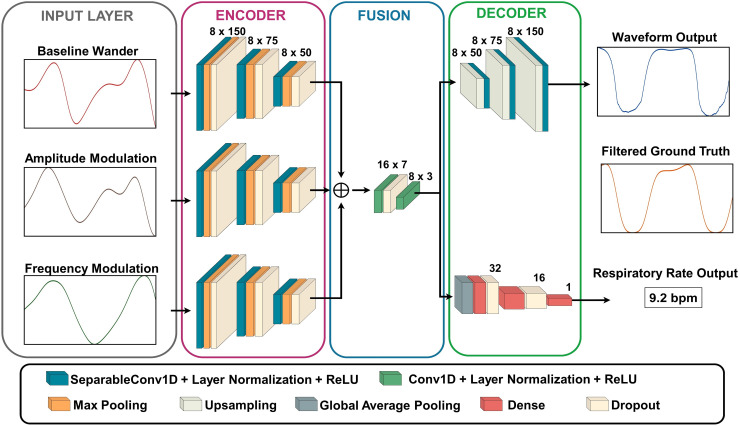
Late fusion multi-task network architecture. Three parallel encoder branches process baseline wander, amplitude modulation, and frequency modulation inputs independently using separable 1D convolutions with kernel sizes of 150, 75, and 50 samples. Encoder outputs are concatenated and processed through a fusion module (16 and 8 kernels). Dual decoder paths produce respiratory waveform reconstruction (via upsampling and separable convolutions) and respiratory rate estimation (via global average pooling and fully connected layers).

The proposed late fusion multi-task architecture comprises three parallel encoder branches, a fusion module, and dual decoder paths for waveform reconstruction and rate estimation (Fig 4). The late fusion strategy enables each respiratory modulation to develop specialized representations before information integration.

Each of the three encoders processes one modulation input (RIIV, RIAV, or RIFV) using an identical architecture of three sequential blocks containing separable 1D convolutions, layer normalization, ReLU activation, max pooling, and dropout (*p* = 0.1). Separable convolutions reduce parameters by approximately 60% compared to standard convolutions, a critical consideration given the limited training data of 37–47 subjects per dataset. Each layer uses 8 kernels, balancing feature extraction capacity against overfitting risk.

The three encoder blocks employ kernel sizes of 150, 75, and 50 samples (corresponding to 5.0, 2.5, and 1.67 seconds at 30 Hz) for multi-scale temporal feature extraction, adopting the symmetric encoder-decoder design principle of the convolutional correlation encoder proposed by Davies and Mandic [39]. The largest kernel captures patterns spanning multiple respiratory cycles. The intermediate kernel focuses on individual cycle characteristics. The smallest captures fine-grained sub-cycle features. Max pooling with stride 2 produces 8-fold temporal reduction across the three blocks, progressively increasing the receptive field.

Encoder outputs are concatenated and processed through a fusion module comprising two convolutional blocks: the first with 16 kernels (kernel size 7, dropout *p* = 0.15) to capture inter-modulation interactions, and the second with 8 kernels (kernel size 3) to compress these relationships into a compact unified representation. This bottleneck design encourages the extraction of salient respiratory information shared across modulations rather than maintaining redundancy.

The waveform decoder reconstructs the filtered ground truth respiratory signal through three upsampling blocks with separable convolutions (kernel sizes 50, 75, 150), layer normalization, and ReLU activation, followed by a final 1D convolution with linear activation. The mirrored kernel progression from 50 to 75–150 samples, which is the reverse of the encoder sequence from 150 to 75–50, enables progressive temporal upsampling that recovers fine-grained waveform structure at each scale. The rate decoder employs global average pooling to aggregate temporal information, followed by two fully connected layers (32 neurons then 16 neurons) with ReLU activation and dropout rates of 0.3 and 0.2, respectively. The higher dropout in the first fully connected layer reflects the greater overfitting risk at larger layer widths, while the lower rate in the narrower second layer allows more stable learning. Sigmoid activation produces normalized respiratory rate in [0,1].

### Training strategies

We designed five training strategies to investigate how clinical context and ground truth modality influence model generalization. Strategy 1 (CapnoBase-only) and Strategy 2 (BIDMC-only) established within-domain baselines by training exclusively on CapnoBase (surgical/anesthesia context, capnography ground truth) and BIDMC (intensive care context, impedance pneumography ground truth), respectively. Strategy 3 (Combined) pooled data from both datasets into a combined training set of 84 subjects, testing whether exposure to heterogeneous data improves generalization or whether the morphological differences between the two ground truth waveforms introduce conflicting optimization objectives. Strategy 4 (Fine-tuned on CapnoBase) pre-trained on BIDMC before fine-tuning on CapnoBase, and Strategy 5 (Fine-tuned on BIDMC) reversed this direction, pre-training on CapnoBase before fine-tuning on BIDMC.

*Subject-level splitting strategy.* Each dataset was partitioned at the subject level into two non-overlapping subsets. Five subjects from CapnoBase and five subjects from BIDMC were withheld as the held-out evaluation set; the remaining 37 subjects (CapnoBase) and 47 subjects (BIDMC) were used for model training across all five strategies. No subject overlap exists between the training and the held-out evaluation sets within either dataset. Owing to the modest cohort size of both benchmarks, a separate validation set was not extracted; early stopping (patience 15 epochs) and learning-rate reduction on plateau (factor 0.5, patience 7 epochs) monitored the segment-level training loss, while dropout in the encoder (*p* = 0.1) and in the rate-decoder fully connected layers (*p* = 0.3 and *p* = 0.2) provided architectural regularization. Two preprocessing configurations were defined based on ground truth filtering: bandpass-filtered at 0.1 to 0.6 Hz versus unfiltered. Combined with the five training strategies, this yielded 10 experimental conditions.

*Optimizer and training schedule.* All models were trained with the Adam optimizer at an initial learning rate of 10^−3^ (default momentum parameters $\beta_{1}=0.9$, $\beta_{2}=0.999$). The global gradient norm was clipped at 1.0 before each parameter update. For the two transfer-learning strategies, the initial learning rate was reduced to 10^−4^ while all other optimizer settings remained identical. An equally weighted Huber loss (δ=0.1) was applied to both decoder outputs (task weights $\lambda_{w}=\lambda_{r}=1$) so that both branches receive balanced gradient signals from the shared encoder. Training ran for a maximum of 100 epochs (batch size 32) with early stopping (patience 15) and learning rate reduction on plateau (factor 0.5, patience 7). Experiments were conducted on a single NVIDIA A100 GPU (40 GB) via Google Colab Pro+.

*Evaluation metrics.* Performance was evaluated using mean absolute error (MAE) and Pearson correlation coefficient (*r*). Waveform MAE quantified pointwise reconstruction error while correlation assessed temporal pattern agreement. For respiratory rate, model outputs were denormalized to breaths per minute before computing MAE. Median values and interquartile ranges across test subjects are reported.

## Results

The proposed framework was able to learn both target outputs from the same short PPG segment: a continuous respiratory waveform and a direct respiratory-rate estimate. The overall pattern of results is summarized in Table 1 and can be interpreted through three main observations. First, sequential transfer learning was the most effective strategy, indicating that knowledge learned from one dataset can support the other when the model is subsequently adapted to the target reference modality. Second, combined training did not improve generalization; instead, it degraded both waveform and rate performance, suggesting that capnography and impedance pneumography impose different waveform reconstruction targets on the shared encoder. Third, bandpass filtering of the reference respiratory waveform generally improved MAE, showing that the definition and preprocessing of the ground-truth signal materially affect multi-task optimization. Under the best transfer-learning configurations, respiratory-rate MAE reached 2.27 bpm on CapnoBase and 1.33 bpm on BIDMC, while waveform MAE reached 19.00% and 20.90%, respectively.

**Table 1:** Results by training strategy and preprocessing configuration. Values are reported as median ± interquartile range (IQR) across held-out subjects. RR MAE values were computed after denormalizing model outputs to breaths per minute. For combined training, metrics were computed on pooled held-out subjects from both datasets.

Strategy	Ground Truth Filter	Waveform MAE (%)	Waveform Corr	RR MAE (bpm)	RR Corr
CapnoBase-only	No Bandpass	25.08 ± 13.66	0.554	2.48 ± 2.43	0.792
	Bandpass	19.56 ± 11.26	0.654	2.32 ± 2.67	0.797
BIDMC-only	No Bandpass	21.14 ± 9.20	0.561	1.40 ± 1.37	0.350
	Bandpass	20.68 ± 8.78	0.600	1.42 ± 1.32	0.404
Combined	No Bandpass	29.78 ± 12.90	0.334	3.20 ± 2.79	0.591
	Bandpass	25.69 ± 9.20	0.425	2.49 ± 2.57	0.722
Fine-tuned on CapnoBase	No Bandpass	22.48 ± 12.57	0.648	2.47 ± 2.61	0.808
	Bandpass	19.00 ± 11.77	0.662	2.27 ± 2.41	0.819
Fine-tuned on BIDMC	No Bandpass	20.59 ± 8.18	0.610	1.48 ± 1.35	0.357
	Bandpass	20.90 ± 8.70	0.591	1.33 ± 1.36	0.417

The following subsections expand these observations in order. Figures are presented for the best-performing transfer-learning configuration on each dataset: BIDMC-to-CapnoBase for CapnoBase evaluation and CapnoBase-to-BIDMC for BIDMC evaluation, both using bandpass-filtered reference waveforms.

### Ground truth filtering improves waveform-target alignment and overall performance

Bandpass filtering of the ground truth signals (0.1 to 0.6 Hz) generally improved model performance, with waveform MAE reductions ranging from 0.5% to 5.5% on most configurations and RR correlation improvements of 0.01 to 0.13. These improvements reflect the removal of high-frequency harmonics present in raw reference signals that are absent from PPG-derived modulations, thereby aligning the optimization target with the frequency content of the input features. However, the effect was not uniform: on BIDMC-only training, filtering slightly increased RR MAE (1.40 to 1.42 bpm) despite improving correlation (0.350 to 0.404), and on CapnoBase-to-BIDMC transfer, waveform MAE marginally increased with filtering (20.59% to 20.90%). These exceptions suggest that the benefit of ground truth filtering depends on the interaction between dataset characteristics and training strategy. Because bandpass-filtered ground truth yielded the strongest overall results on 8 of 10 metrics, results discussed below refer to this configuration unless stated otherwise.

### Single-dataset baseline performance

Models trained exclusively on CapnoBase achieved respiratory rate MAE of 2.32 ± 2.67 bpm (*r* = 0.797) with waveform reconstruction MAE of 19.56 ± 11.26% (*r* = 0.654). On BIDMC, single-dataset training produced respiratory rate MAE of 1.42 ± 1.32 bpm (*r* = 0.404) and waveform reconstruction MAE of 20.68 ± 8.78% (*r* = 0.600). The lower respiratory rate correlation on BIDMC reflects the narrower rate distribution in this adult intensive care population, which compresses the dynamic range available for correlation estimation; arterial blood pressure oscillations near 0.1 Hz in ICU recordings further complicate respiratory frequency extraction.

### Ground truth mismatch degrades multi-task performance under combined training

Pooling data from both datasets into a single training set degraded performance relative to these single-dataset baselines on every metric. The best combined configuration achieved respiratory rate MAE of 2.49 ± 2.57 bpm (*r* = 0.722), an increase of 0.17 bpm over CapnoBase-only and 1.07 bpm over BIDMC-only training, with waveform reconstruction MAE of 25.69 ± 9.20% (*r* = 0.425). This degradation stems from the structural mismatch between the two ground truth modalities: capnography produces sharp waveform transitions at breath boundaries, while impedance pneumography generates smoother sinusoidal patterns tracking chest wall movement. The waveform decoder cannot converge on a single representation that accommodates both morphologies, and the resulting conflict propagates through the shared encoder features to impair rate estimation as well.

### Transfer learning outperforms combined training

Transfer learning through sequential domain adaptation yielded the strongest results across both datasets. Pre-training on BIDMC followed by CapnoBase fine-tuning achieved respiratory rate MAE of 2.27 ± 2.41 bpm with the highest correlation observed across all strategies (*r* = 0.819), and waveform reconstruction MAE of 19.00 ± 11.77% (*r* = 0.662). In the reverse direction, pre-training on CapnoBase followed by BIDMC fine-tuning achieved respiratory rate MAE of 1.33 ± 1.36 bpm (*r* = 0.417) and waveform reconstruction MAE of 20.90 ± 8.70% (*r* = 0.591), improving over the BIDMC-only rate estimation baseline by 0.09 bpm while maintaining comparable waveform fidelity. Both transfer directions produced lower respiratory rate MAE than combined training by 0.22 to 1.16 bpm, indicating that fine-tuning preserves modality-specific waveform reconstruction capabilities while adapting rate estimation features to the target domain.

### Waveform reconstruction quality

**Fig 5:**
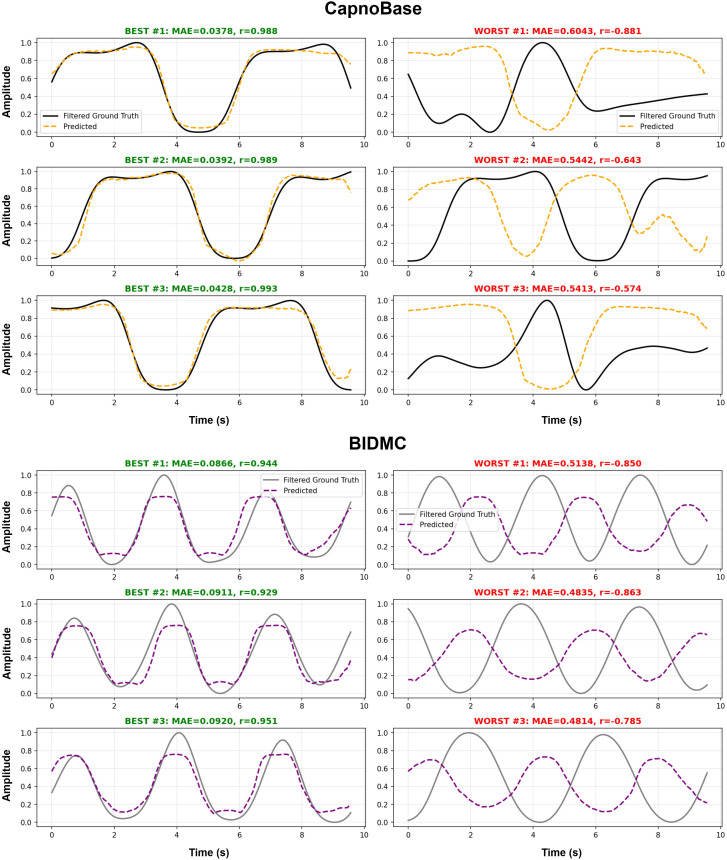
Waveform reconstruction quality comparison showing the three best and worst performing samples for CapnoBase and BIDMC datasets. Upper panel: CapnoBase results from BIDMC-to-CapnoBase transfer learning (Strategy 4, Bandpass). Lower panel: BIDMC results from CapnoBase-to-BIDMC transfer learning (Strategy 5, Bandpass). Best samples (left columns) show MAE below 4 to 9% with correlations exceeding 0.90. Worst samples (right columns) exhibit MAE above 48 to 60% with negative correlations and phase shifts, though dominant respiratory frequency is preserved.

Fig 5 presents representative waveform reconstructions from the transfer learning configurations, contrasting the three highest-quality and lowest-quality samples for each dataset. For CapnoBase, the best reconstructions achieved MAE of 3.78 to 4.28% with correlations exceeding 0.98 (*r* from 0.988 to 0.993), accurately capturing the sharp inspiration-expiration transitions and plateau phases characteristic of capnography. The worst cases exhibited MAE of 54–60% with strongly negative correlations (r=−0.574 to −0.881), indicating that the predicted waveforms were approximately anti-phase relative to the ground truth. Visual inspection confirms that predicted peaks align with ground truth troughs in these segments, suggesting the model inverts the respiratory phase under conditions of ambiguous or low signal-to-noise input features; however, the dominant respiratory frequency was preserved, which explains why rate estimation remained accurate even when waveform fidelity was poor. For BIDMC, the best reconstructions reached MAE of 8.66 to 9.20% with correlations exceeding 0.92 (*r* from 0.929 to 0.951), reproducing the smoother sinusoidal morphology of impedance pneumography, while the worst cases showed MAE of 48–51% with negative correlations (r=−0.785 to −0.863), exhibiting a similar anti-phase pattern. Despite this phase inversion in the worst cases, the oscillation frequency of the predicted waveforms matched the filtered ground truth, explaining the preservation of rate estimation accuracy.

**Fig 6:**
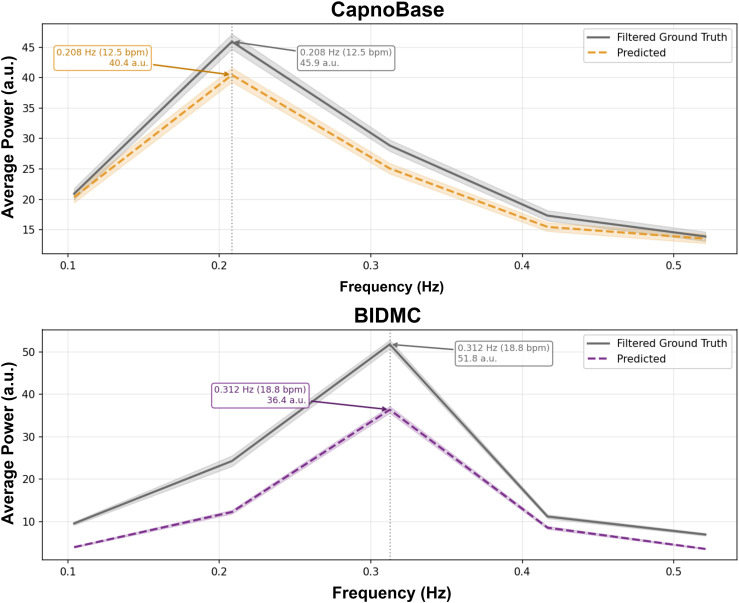
Average power spectrum comparison between filtered ground truth and predicted respiratory signals for CapnoBase and BIDMC. Upper panel: CapnoBase showing dominant peak at 0.208 Hz (12.5 bpm). Lower panel: BIDMC showing a peak near 0.312 Hz (18.8 bpm). Shaded regions represent ±1 standard deviation. Predicted spectra preserve dominant frequency content with reduced overall power.

Spectral analysis (Fig 6) confirms that predicted waveforms preserve the dominant respiratory frequency on both datasets. On CapnoBase, the dominant spectral peak at 0.208 Hz (12.5 bpm) is accurately reconstructed in the predicted spectra, with close agreement in peak location and shape. The predicted spectra show slightly lower power compared to the filtered ground truth (approximately 40.4 versus 45.9 a.u. at the peak), with this power gap widening progressively from 0.208 to 0.4 Hz before both curves converge near 0.5 Hz. This pattern suggests that the model produces smoothed respiratory representations that attenuate higher-frequency components and noise while retaining the fundamental breathing frequency. On BIDMC, the dominant peak appears near 0.312 Hz (18.8 bpm) as a sharper spectral feature, reflecting the more uniform rate distribution in this adult population. The predicted spectra exhibit substantially lower overall power than the filtered ground truth across the entire respiratory band (approximately 36.4 versus 51.8 a.u. at the peak), which is consistent with the amplitude underestimation observed in the worst-case waveform reconstructions. Despite this power reduction, the peak location is accurately preserved, confirming that the model captures the correct frequency content even when amplitude fidelity is reduced.

### Respiratory rate prediction performance

**Fig 7:**
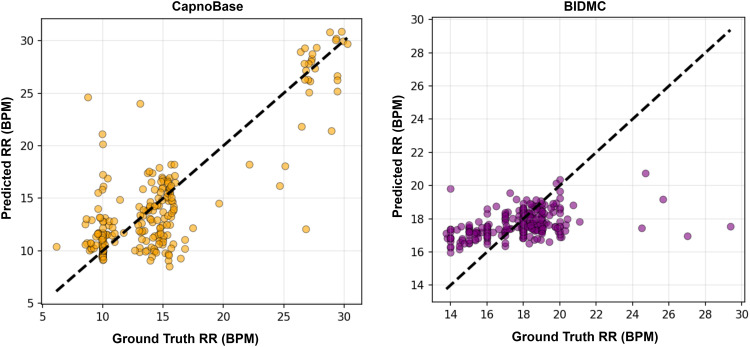
Respiratory rate prediction scatter plots comparing predicted values against ground truth for CapnoBase and BIDMC. Left panel: CapnoBase (Strategy 4, Bandpass). Right panel: BIDMC (Strategy 5, Bandpass). Dashed lines indicate perfect agreement. CapnoBase shows broad agreement across 6 to 31 bpm with greater scatter in the mid-range; BIDMC shows tight clustering at 16 to 20 bpm with underestimation at higher rates.

Fig 7 shows scatter plots of predicted versus ground truth respiratory rates. For CapnoBase, predictions follow the identity line across a wide range (6–31 bpm), reflecting the pediatric-adult mix in this dataset. Points cluster densely in the 9–16 bpm range corresponding to the pediatric subpopulation, with a secondary cluster at 27–31 bpm that adheres more closely to the identity line. Deviation from the identity line is more pronounced in the mid-range, particularly at lower rates. For BIDMC, predictions cluster tightly within 16–20 bpm, consistent with the narrower rate distribution in this adult ICU population. At ground truth rates above 22 bpm, predicted values tend to fall below the identity line, likely reflecting a regression-toward-the-mean effect at the distribution tails. Neither dataset shows a systematic directional bias overall.

**Fig 8:**
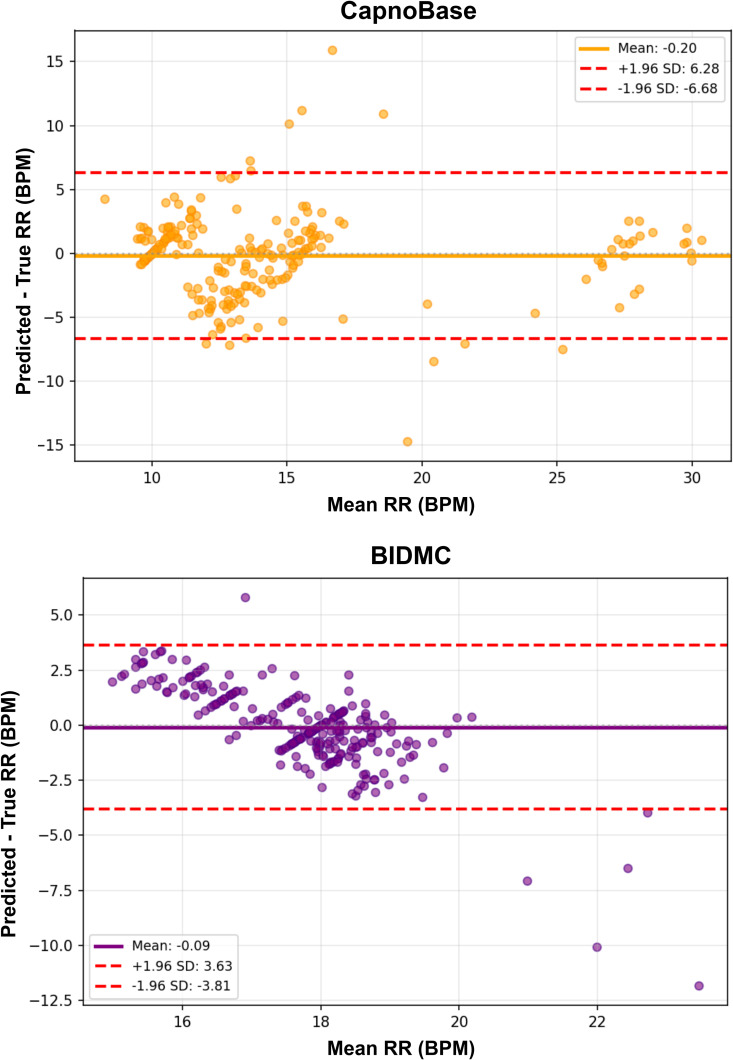
Bland-Altman plots showing agreement between predicted and ground truth respiratory rates for CapnoBase and BIDMC. Upper panel: CapnoBase (mean bias: −0.20 bpm; 95% limits of agreement: −6.68 to 6.28 bpm). Lower panel: BIDMC (mean bias: −0.09 bpm; 95% limits of agreement: −3.81 to 3.63 bpm). Both datasets show negligible mean bias with symmetric error distributions; isolated outliers at higher mean rates on BIDMC reflect regression toward the training population mean.

Bland-Altman analysis (Fig 8) reveals mean bias of −0.20 bpm (95% limits of agreement: −6.68 to 6.28 bpm) on CapnoBase and −0.09 bpm (95% limits: −3.81 to 3.63 bpm) on BIDMC. On CapnoBase, data points are distributed symmetrically around the zero-bias line across the 8–30 bpm range, with most errors falling within ±5 bpm; a single extreme outlier near mean rate 19 bpm shows an error of approximately −14.5 bpm. On BIDMC, the error distribution is concentrated within ±2 bpm for mean rates between 15 and 20 bpm; however, isolated outliers at mean rates above 20 bpm exhibit large negative errors (up to −10 to −12 bpm), reflecting the regression-toward-the-mean behavior observed in the scatter analysis. These outliers correspond to segments where the ground truth rate exceeds the dominant population range, and the model’s predictions regress toward the training distribution mean. Despite these outliers, both datasets show negligible mean bias, and the error distribution remains symmetric around zero for the majority of observations.

## Discussion

This study introduced a late fusion multi-task architecture that leverages physiologically grounded RIIV, RIAV, and RIFV features for simultaneous respiratory waveform reconstruction and rate estimation from PPG.

### Architectural design and multi-task performance

The proposed architecture jointly delivers waveform reconstruction and rate estimation, with the per-condition metrics reported in Table 1. These results compare favorably with recent deep learning approaches while offering improved interpretability through the explicit use of RIIV, RIAV, and RIFV as structured inputs. Building on the approach of Lampier et al. [36], who first demonstrated the benefit of feeding these modulations into a deep network for rate estimation, our design ensures that the features entering the network correspond to known respiratory-cardiovascular coupling mechanisms rather than opaque learned representations.

A key architectural advantage of this framework over existing approaches is the dedicated rate decoder branch. The corr-encoder of Davies and Mandic [39], which pioneered the convolutional encoder-decoder paradigm for PPG-to-respiratory waveform reconstruction, achieved a median waveform MAE of 27% on CapnoBase using 9.6-second windows under leave-one-subject-out cross-validation and a single-task architecture with raw PPG input. To obtain respiratory rate from the reconstructed waveform, Davies and Mandic applied post-hoc FFT, which required extending the evaluation window to 30.6 seconds to achieve sufficient spectral resolution; this yielded median absolute errors of 0.37 bpm on CapnoBase and 0.89 bpm on BIDMC after retraining. Our multi-task architecture provides rate estimates directly from the rate decoder without post-hoc processing, and does so within the same 9.6-second window used for waveform reconstruction. This capability is clinically relevant, as shorter estimation windows enable faster detection of respiratory rate changes, particularly in scenarios such as pediatric anesthesia where breathing rate can shift rapidly.

Regarding waveform reconstruction, the best-configuration MAE of 19.00% on CapnoBase improves upon the 27% median MAE reported by Davies and Mandic [39]. This improvement likely reflects two factors: the use of physiologically decomposed modulation inputs (RIIV, RIAV, RIFV) rather than raw PPG, which provides the encoder with pre-separated respiratory components; and the multi-task training signal from the rate decoder, which may regularize the shared encoder toward representations that better preserve respiratory periodicity. However, direct numerical comparison should be interpreted cautiously, as the evaluation protocols differ (5 held-out test subjects versus leave-one-subject-out cross-validation in [39]).

The multi-task formulation provides a theoretical advantage that the results partially support. By jointly optimizing waveform reconstruction and rate estimation, the shared encoder must learn representations that serve both tasks. The observation that rate estimation remained accurate even when waveform fidelity was poor, because the dominant respiratory frequency was preserved despite amplitude and phase errors, suggests that the two decoders extract complementary information from the shared representation. This complementarity is the condition under which multi-task learning provides benefits over single-task alternatives [50].

### Transfer learning outperforms combined training

Perhaps the most practically significant finding is that combined training, in which data from datasets with different reference modalities were pooled, consistently degraded performance relative to both single-dataset training and transfer learning. This degradation was most pronounced in waveform reconstruction (correlation dropping to 0.33 to 0.43) but also propagated to rate estimation (correlation 0.59 to 0.72), confirming that the conflict between capnography’s sharp transitions and impedance pneumography’s sinusoidal patterns affects the entire multi-task pipeline through shared encoder representations.

Transfer learning circumvents this limitation by exposing the model to different modalities sequentially rather than simultaneously. During pre-training, the encoder develops general respiratory feature representations from the source domain. During fine-tuning, the waveform decoder specializes for the target modality while the encoder adapts incrementally, preserving useful features learned in the first stage. The two transfer directions yielded different outcomes: the BIDMC-to-CapnoBase direction produced the highest rate correlation across all strategies, while the CapnoBase-to-BIDMC direction achieved the lowest rate MAE on BIDMC (Table 1). This asymmetry likely reflects the broader rate distribution in CapnoBase (5–48 bpm versus 5–34 bpm in BIDMC), which provides a richer dynamic range for correlation estimation, and the greater challenge of adapting from smoother impedance pneumography waveforms to sharper capnography transitions.

Interestingly, Davies and Mandic [39] observed a similar benefit from cross-dataset training: their corr-encoder trained exclusively on BIDMC produced worse results than the model pre-trained on CapnoBase then retrained on BIDMC, suggesting that CapnoBase contains richer respiratory information that benefits generalization. Our transfer learning results corroborate this observation within a multi-task context and further reveal that the direction of transfer matters when ground truth modalities differ structurally.

These observations carry broader implications. As multi-task architectures become increasingly popular for physiological signal analysis, researchers should carefully consider whether pooling datasets with heterogeneous reference standards will benefit or degrade model performance. Our results suggest that when the objective includes waveform reconstruction with structurally different ground truth, data pooling is counterproductive, and transfer learning offers a principled alternative.

### Potential clinical translation

We see three clinical contexts where this framework could be useful, though prospective validation is needed before deployment in any of them. During pediatric anesthesia, from which the CapnoBase recordings originate [16,51], capnography is standard but often interrupted at mask induction and emergence; a PPG sensor already in place for pulse oximetry could provide continuous respiratory estimates at precisely these moments. In adult intensive care, represented by the BIDMC patients [32,52], movement and electrode repositioning often disrupt impedance pneumography traces, and the same PPG signal could fill these monitoring gaps. On general wards, respiratory rate remains the vital sign least reliably recorded in routine nursing rounds [4], and a wearable PPG device running this framework could deliver the continuous respiratory data that NEWS scoring [3] needs but rarely receives.

### Comparison with existing methods

Comparing results across studies is complicated by differences in evaluation protocols, test set composition, and subject overlap; Table 2 is therefore presented as a contextual reference rather than a strict ranking, with protocol details noted in the Method column. Among the studies listed, this work is the only one to report both waveform-reconstruction and respiratory-rate metrics on both CapnoBase and BIDMC; it also operates on the shortest analysis window of any approach that jointly delivers both outputs: 9.6 seconds, compared with the 30.6-second window that Davies and Mandic require for post-hoc rate extraction and the 7- to 64-second windows of the rate-only methods.

**Table 2:** Protocol-aware comparison of respiratory-rate and waveform reconstruction performance across representative PPG-based deep learning studies and the present work, on the CapnoBase and BIDMC benchmarks. Error statistics: mean AE unless indicated otherwise. ${\;}^{a}$Values reported by Chin et al. after removal of 12 segments with severe signal fluctuations. A dash (-) indicates a metric not reported by the respective study. Studies differ in window length, training protocol, and post-processing; numerical values are not directly comparable across rows.

Study	Model	Dataset	Window (s)	RR MAE (bpm)	Waveform MAE (%)	Method
Davies and Mandic [39]	Corr-encoder	CapnoBase	9.6	–	27 (median)	Waveform evaluation; LOSO cross-validation
			30.6	0.37 (median AE)	–	Rate by post-hoc FFT; LOSO cross-validation
		BIDMC	30.6	0.89 (median AE)	–	CapnoBase pretrained, BIDMC fine-tuned; LOSO
Chin et al. [44]	CNN-LSTM	BIDMC	7	2.42 (2.02${\;}^{a}$)	–	50/50 train-test split on BIDMC
		CapnoBase (BIDMC-pretrained)	7	1.99 (1.24${\;}^{a}$)	–	Trained on BIDMC only, tested on CapnoBase
Yang et al. [45]	Spiking neural network	BIDMC	16	1.37	–	13-subject test set on BIDMC
			32	1.22	–	
			64	1.15	–	
**Present work**	**Late Fusion Multi-Task**	**CapnoBase**	**9.6**	**2.27** (r=0.819)	**19.00** (r=0.662)	**BIDMC pretrained, CapnoBase fine-tuned; 5-subject held-out test**
		**BIDMC**	**9.6**	**1.33** (r=0.417)	**20.90** (r=0.591)	**CapnoBase pretrained, BIDMC fine-tuned; 5-subject held-out test**

The waveform-reconstruction MAE of 27% reported by Davies and Mandic on CapnoBase, evaluated on 9.6-second windows under leave-one-subject-out cross-validation, is the only published comparator for the present waveform results. The comparison should be interpreted cautiously because the two values are computed under different evaluation protocols (median versus mean error; leave-one-subject-out versus subject-level held-out). With that protocol caveat, this framework achieves comparable waveform performance while also providing a direct respiratory-rate output and using a window roughly one-third as long.

**Respiratory rate estimation.** The corr-encoder of Davies and Mandic [39] reports lower rate errors on both datasets, but three methodological differences bear on the comparison. First, their overlapping-segment-and-averaging pipeline systematically reduces prediction variance relative to evaluating individual segments as we do. Second, they report median absolute error, which yields lower values than mean absolute error when the error distribution is right-skewed. Third, respiratory rate is extracted via FFT on the smoothed output waveform rather than by direct regression. Chin et al. [44] used a CNN-LSTM model with 7-second windows, reporting MAE of 2.42 bpm on BIDMC (50:50 train-test split) and 1.99 bpm on CapnoBase as a cross-dataset test (model trained on BIDMC). After removing 12 segments with severe signal fluctuations, these errors improved to 2.02 and 1.24 bpm respectively. Yang et al. [45] achieved MAE of 1.37, 1.22, and 1.15 bpm on BIDMC using window sizes of 16, 32, and 64 seconds respectively, which are substantially longer than our 9.6 second window. Their test set comprised 13 subjects; however, their evaluation was limited to BIDMC, where the narrow rate distribution (14–20 bpm) may favor estimation.

**Waveform reconstruction.** Despite the protocol differences noted above, our mean waveform MAE of 19.00% on CapnoBase compares favorably with the median of 27% reported by Davies and Mandic [39]. This improvement is likely attributable to the use of physiologically decomposed modulation inputs rather than raw PPG, and to the multi-task training signal from the rate decoder, which may regularize the encoder toward representations preserving respiratory periodicity. Shuzan et al. [40] reported substantially higher waveform correlations (*r* from 0.94 to 0.96) with their dedicated PPG2RespNet architecture; however, their model was specifically optimized for waveform reconstruction as a single task without rate estimation, and their evaluation protocol differs from ours.

**Multi-task comparison.** An architectural distinction of this work is the joint waveform and rate output. Most published methods, including Chin et al. [44] and Yang et al. [45], estimate rate as a scalar without producing the underlying waveform. Our dual-decoder design provides richer clinical information within a single forward pass. Relative to the most closely related multi-task work by Feli et al. [50], which jointly estimated heart rate and respiratory rate from smartwatch PPG data during daily activities, our architecture addresses a different and arguably more demanding multi-task formulation: joint waveform reconstruction and rate estimation. Waveform reconstruction requires predicting a high-dimensional output sequence rather than a scalar, imposing substantially greater demands on the shared feature representation. That our rate estimation performance remains competitive alongside meaningful waveform reconstruction demonstrates the feasibility of this more ambitious multi-task objective.

### Limitations and future work

We observed phase-inverted waveform reconstructions in a minority of segments, where predicted peaks aligned with ground truth troughs despite the dominant respiratory frequency being preserved; this may reflect the inability of a pointwise loss to capture temporal alignment, suggesting that refinements of the loss function or polarity correction may improve reconstruction consistency. All evaluations were conducted on established benchmark datasets under controlled recording conditions, and validating the complete pipeline on wearable devices under real-world ambulatory conditions remains the key next step.

## Conclusion

We presented a late fusion multi-task architecture that extracts baseline wander, amplitude modulation, and frequency modulation from PPG signals for simultaneous respiratory waveform reconstruction and rate estimation. This approach embeds established respiratory-cardiovascular coupling mechanisms into the network design, enabling interpretable feature extraction while maintaining computational efficiency through separable convolutions. A dedicated rate decoder branch provides direct respiratory rate estimates without the post-hoc spectral analysis required by single-task waveform approaches, enabling clinically relevant estimation from 9.6-second windows, roughly one-third the length needed by prior methods.

The architecture achieved waveform reconstruction MAE of 19.00% (*r* = 0.662) and respiratory rate MAE of 2.27 bpm (*r* = 0.819) on CapnoBase, and waveform MAE of 20.90% (*r* = 0.591) and respiratory rate MAE of 1.33 bpm (*r* = 0.417) on BIDMC. Notably, different ground truth modalities, capnography’s sharp breath transitions versus impedance pneumography’s smooth sinusoidal patterns, create conflicting objectives when training a single model to reconstruct both waveform types simultaneously. Combined training degraded respiratory rate estimation by 0.22 to 1.16 bpm compared to transfer learning, indicating that datasets with different reference modalities should not be pooled for multi-task waveform reconstruction. Transfer learning addresses this limitation by first learning one modality, then fine-tuning for another. These findings highlight the need for careful consideration of reference signal characteristics when designing multi-task respiratory monitoring systems.
