# Supplementary material for: A late fusion multi-task learning for respiratory waveform and rate estimation from photoplethysmography
Source: PLoS One. 2026 Jul 10;21(7):e0353203. doi: 10.1371/journal.pone.0353203 (PMC13353955; doi:10.1371/journal.pone.0353203)
Supplement: Attachment — Submitted filename: Response_to_Reviewers.docx [file pone.0353203.s001.docx]

**RESPONSES TO REVIEWERS' COMMENTS**

**Manuscript:** PONE-D-26-10284

**Title:** *"A late fusion multi-task learning for respiratory waveform and rate estimation from photoplethysmography"*

**Journal:** PLOS ONE

**PLEASE NOTE:** Reviewer comments are *italicised*. Our replies follow in normal font. Text shown in red within the **"Changes made in the manuscript"** sections indicates the specific portions that were modified relative to the original manuscript. Page and line numbers refer to the revised manuscript.

**********************************************************************************

**Reviewer 1**

**Comment 1.**

*"Strengthen the abstract to give a clear summary, strong contribution, and quantitative results to emphasize relevance and key results only."*

**Reply:**

We thank the reviewer for the constructive suggestion. The Abstract has been revised to clarify the methodological contribution (single forward pass, 9.6-second window, dual task-specific decoders), to specify the dataset sizes (n = 42 and n = 52), and to emphasise reference signal compatibility in the closing sentence. The quantitative results have been retained.

**Changes made in the manuscript**

**Page 1**

Continuous respiratory monitoring enables early detection of physiological deterioration, yet conventional capnography remains impractical for prolonged use. Photoplethysmography (PPG) offers a non-invasive alternative that encodes respiratory information through baseline wander (respiratory-induced intensity variation; RIIV), amplitude modulation (respiratory-induced amplitude variation; RIAV), and frequency modulation (respiratory-induced frequency variation; RIFV) of the pulsatile waveform. Existing PPG-based deep learning approaches, whether operating on the raw signal or on these physiological modulations, are limited to single-task architectures that estimate either respiratory rate or reconstruct the respiratory waveform in isolation, without jointly addressing both outputs. We propose a late fusion multi-task framework in which dedicated encoder branches independently process each modulation before fusion, and dual decoders simultaneously reconstruct the respiratory waveform and estimate the respiratory rate. The framework was evaluated on the CapnoBase (n = 42) and BIDMC (n = 52) benchmarks across multiple training strategies. For respiratory-rate estimation, the best transfer-learning configurations achieved MAE of 2.27 bpm on CapnoBase and 1.33 bpm on BIDMC. For waveform reconstruction, the corresponding MAE values were 19.00% and 20.90%, with moderate correlations (r = 0.662 and r = 0.591, respectively). Sequential transfer learning consistently outperformed all other strategies, whereas pooled training degraded both outputs, demonstrating that capnography-derived and impedance-derived waveforms are not interchangeable training targets. These findings establish that short-window PPG can simultaneously support respiratory-rate estimation and waveform reconstruction, when reference signal compatibility is explicitly addressed in multi-task training.

**Comment 2.**

*"Same goes for the introduction section, I would recommend to streamline and reduce redundancy, and move the physiological detail to Methods."*

**Reply:**

We thank the reviewer for the helpful recommendation. The paragraph detailing the physiological mechanisms of RIIV, RIAV, and RIFV has been moved to the Signal preprocessing subsection of the Methods. The Introduction now retains only the three modulation names and a pointer to the Methods section.

**Changes made in the manuscript**

**Page 2 (Lines 24-30)**

These mechanisms arise from the coupling between respiratory and cardiovascular systems [15, 16]. Three main modulation pathways have been identified and their time-frequency signatures characterized [17]: respiratory-induced intensity variation (RIIV, also termed baseline wander, BW) [18, 19], respiratory-induced amplitude variation (RIAV, also termed amplitude modulation, AM) [20], and respiratory-induced frequency variation (RIFV, also termed frequency modulation, FM) [21, 22]. The physiological mechanisms underlying each modulation are described in detail in the Methods section. Together, these modulations form the physiological basis for extracting both respiratory waveforms and rate from PPG signals.

**Page 4 (Lines 116-127)**

Three physiological mechanisms couple respiration to the PPG waveform [15, 16]. Baseline wander (BW), or respiratory-induced intensity variation (RIIV), reflects respiratory modulation of venous return and tissue blood volume: during inspiration, decreasing intrathoracic pressure increases venous return to the right heart while reducing peripheral venous volume, producing slow baseline oscillations at the respiratory frequency [18]. The influence of different ventilation modes on this component has been characterized under controlled conditions [19]. Amplitude modulation (AM), or respiratory-induced amplitude variation (RIAV), originates from cyclic variations in stroke volume across the breathing cycle, which modulate pulse-wave amplitude [20]. Frequency modulation (FM), or respiratory-induced frequency variation (RIFV), manifests as respiratory sinus arrhythmia, a beat-to-beat heart rate fluctuation driven by parasympathetic modulation of cardiac pacing [21, 22] and thought to facilitate pulmonary gas exchange. All three modulations operate within the respiratory frequency band (0.1 to 0.6 Hz) and are therefore separable from cardiac components (0.8 to 3.0 Hz) through bandpass filtering and dedicated extraction algorithms described below.

**References**

**15.** Chen M, Zhu Q, Wu M, Wang Q. Modulation model of the photoplethysmography signal for vital sign extraction. *IEEE J Biomed Health Inform.* 2021;25(4):969–77. doi:10.1109/JBHI.2020.3013811.

**16.** Karlen W, Raman S, Ansermino JM, Dumont GA. Multiparameter respiratory rate estimation from the photoplethysmogram. *IEEE Trans Biomed Eng.* 2013;60(7):1946–53. doi:10.1109/TBME.2013.2246160.

**17.** Cicone A, Wu HT. How nonlinear-type time-frequency analysis can help in sensing instantaneous heart rate and instantaneous respiratory rate from photoplethysmography in a reliable way. *Front Physiol.* 2017;8:701. doi:10.3389/fphys.2017.00701.

**18.** Meredith DJ, Clifton D, Charlton P, Brooks J, Pugh CW, Tarassenko L. Photoplethysmographic derivation of respiratory rate: A review of relevant physiology. *J Med Eng Technol.* 2012;36(1):1–7. doi:10.3109/03091902.2011.638965.

**19.** Alian AA, Shelley KH. Respiratory physiology and the impact of different modes of ventilation on the photoplethysmographic waveform. *Sensors (Basel).* 2012;12(2):2236–54. doi:10.3390/s120202236.

**20.** Johansson A, Öberg PÅ. Estimation of respiratory volumes from the photoplethysmographic signal. Part 2: A model study. *Med Biol Eng Comput.* 1999;37(1):48–53. doi:10.1007/BF02513265.

**21.** Penzel T, Kantelhardt JW, Bartsch RP, Riedl M, Kraemer JF, Wessel N, et al. Modulations of heart rate, ECG, and cardio-respiratory coupling observed in polysomnography. *Front Physiol.* 2016;7:460. doi:10.3389/fphys.2016.00460.

**22.** Hayano J, Yasuma F. Hypothesis: Respiratory sinus arrhythmia is an intrinsic resting function of cardiopulmonary system. *Cardiovasc Res.* 2003;58(1):1–9. doi:10.1016/S0008-6363(02)00851-9.

**Comment 3.**

*"For the Results chapter I would recommend to summarize key findings first, then elaborate."*

**Reply:**

We thank the reviewer for this valuable suggestion. We have rewritten the opening of the Results section so that readers first see the major findings before reviewing the full table. The revised section now introduces the results in a clear sequence: transfer learning performance, the limitation of combined training across heterogeneous reference modalities, and the effect of ground-truth filtering.

**Changes made in the manuscript**

**Pages 6-7 (Lines 215-229)**

The proposed framework was able to learn both target outputs from the same short PPG segment: a continuous respiratory waveform and a direct respiratory-rate estimate. The overall pattern of results is summarized in Table 1 and can be interpreted through three main observations. First, sequential transfer learning was the most effective strategy, indicating that knowledge learned from one dataset can support the other when the model is subsequently adapted to the target reference modality. Second, combined training did not improve generalization; instead, it degraded both waveform and rate performance, suggesting that capnography and impedance pneumography impose different waveform reconstruction targets on the shared encoder. Third, bandpass filtering of the reference respiratory waveform generally improved MAE, showing that the definition and preprocessing of the ground-truth signal materially affect multi-task optimization. Under the best transfer-learning configurations, respiratory-rate MAE reached 2.27 bpm on CapnoBase and 1.33 bpm on BIDMC, while waveform MAE reached 19.00% and 20.90%, respectively.

The following subsections expand these observations in order. Figures are presented for the best-performing transfer-learning configuration on each dataset: BIDMC-to-CapnoBase for CapnoBase evaluation and CapnoBase-to-BIDMC for BIDMC evaluation, both using bandpass-filtered reference waveforms.

**Comment 4.**

*"In the Discussion section limitations and future work could be included."*

**Reply:**

We thank the reviewer for highlighting this point. A Limitations and Future Work subsection has been added at the end of the Discussion, covering phase-inverted waveform reconstructions and the scope of current evaluations, along with plans for real-world validation on wearable devices.

**Changes made in the manuscript**

**Page 13 (Lines 442-448)**

**Limitations and Future Work**

We observed phase-inverted waveform reconstructions in a minority of segments, where predicted peaks aligned with ground truth troughs despite the dominant respiratory frequency being preserved; this may reflect the inability of a pointwise loss to capture temporal alignment, suggesting that refinements of the loss function or polarity correction may improve reconstruction consistency. All evaluations were conducted on established benchmark datasets under controlled recording conditions, and validating the complete pipeline on wearable devices under real-world ambulatory conditions remains the key next step.

**Comment 5.**

*"Repeating metrics already presented could be avoided."*

**Reply:**

We thank the reviewer for this useful observation. In the Discussion, the opening paragraph of Architectural Design and Multi-Task Performance has been rewritten to point to Table 1 rather than restate the four MAE values, and the former Ground Truth Filtering Improves Overall Performance subsection has been removed, since the corresponding finding is already given once in the Results section.

**Changes made in the manuscript**

**Page 10 (Lines 327-333)**

The proposed architecture jointly delivers waveform reconstruction and rate estimation, with the per-condition metrics reported in Table 1. These results compare favorably with recent deep learning approaches while offering improved interpretability through the explicit use of RIIV, RIAV, and RIFV as structured inputs. Building on the approach of Lampier et al. [36], who first demonstrated the benefit of feeding these modulations into a deep network for rate estimation, our design ensures that the features entering the network correspond to known respiratory-cardiovascular coupling mechanisms rather than opaque learned representations.

**Reviewer 2**

**Comment 1.**

*"It is suggested to add a concise comparison table in the 'Comparison with existing methods' section, which summarizes the optimal results of this study and the core metrics (e.g., respiratory rate MAE, waveform MAE/correlation) of recent representative studies such as Davies and Mandic (2023) and Chin et al. (2024) on the CapnoBase and BIDMC datasets, so as to intuitively reflect the performance positioning."*

**Reply:**

We thank the reviewer for the useful suggestion. A new Table 2 has been added to the Comparison with Existing Methods subsection, summarising three representative studies (Davies and Mandic 2023 [39], Chin et al. 2024 [44], and Yang et al. 2024 [45]) and the present work on CapnoBase and BIDMC. The paragraph above the table draws out the two main observations, and the paragraph that follows addresses the protocol caveats for the waveform comparison.

**Changes made in the manuscript**

**Page 11-12 (Lines 398-411)**

Comparing results across studies is complicated by differences in evaluation protocols, test set composition, and subject overlap; Table 2 is therefore presented as a contextual reference rather than a strict ranking, with protocol details noted in the Method column. Among the studies listed, this work is the only one to report both waveform-reconstruction and respiratory-rate metrics on both CapnoBase and BIDMC; it also operates on the shortest analysis window of any approach that jointly delivers both outputs: 9.6 seconds, compared with the 30.6-second window that Davies and Mandic require for post-hoc rate extraction and the 7- to 64-second windows of the rate-only methods.

**Table 2. Protocol-aware comparison of respiratory-rate and waveform reconstruction performance across representative PPG-based deep learning studies and the present work, on the CapnoBase and BIDMC benchmarks**. Error statistics: mean AE unless indicated otherwise. ^a^Values reported by Chin et al. after removal of 12 segments with severe signal fluctuations. A dash (-) indicates a metric not reported by the respective study. Studies differ in window length, training protocol, and post-processing; numerical values are not directly comparable across rows.

| **Study** | **Model** | **Dataset** | **Window (s)** | **RR MAE (bpm)** | **Waveform MAE (%)** | **Method** |
| --- | --- | --- | --- | --- | --- | --- |
| Davies and Mandic [39] | Corr-encoder | CapnoBase | 9.6 | - | 27 (median) | Waveform evaluation; LOSO cross-validation |
|  |  |  | 30.6 | 0.37 (median AE) | - | Rate by post-hoc FFT; LOSO cross-validation |
|  |  | BIDMC | 30.6 | 0.89 (median AE) | - | CapnoBase pretrained, BIDMC fine-tuned; LOSO |
| Chin et al. [44] | CNN-LSTM | BIDMC | 7 | 2.42 (2.02^a^) | - | 50/50 train-test split on BIDMC |
|  |  | CapnoBase (BIDMC-pretrained) | 7 | 1.99 (1.24^a^) | - | Trained on BIDMC only, tested on CapnoBase |
| Yang et al. [45] | Spiking neural network | BIDMC | 16 | 1.37 | - | 13-subject test set on BIDMC |
|  |  |  | 32 | 1.22 | - |  |
|  |  |  | 64 | 1.15 | - |  |
| **Present work** | **Late Fusion Multi-Task** | **CapnoBase** | **9.6** | **2.27 (r = 0.819)** | **19.00 (r = 0.662)** | **BIDMC pretrained, CapnoBase fine-tuned; 5-subject held-out test** |
|  |  | **BIDMC** | **9.6** | **1.33 (r = 0.417)** | **20.90 (r = 0.591)** | **CapnoBase pretrained, BIDMC fine-tuned; 5-subject held-out test** |

The waveform-reconstruction MAE of 27% reported by Davies and Mandic on CapnoBase under leave-one-subject-out cross-validation is the only published comparator for the present waveform results, and the comparison should be interpreted cautiously because the two values are computed under different evaluation protocols (median versus mean error; leave-one-subject-out versus subject-level held-out). With that protocol caveat, this framework achieves comparable waveform performance while also providing a direct respiratory-rate output and using a window roughly one-third as long.

**References**

**39**. Davies HJ, Mandic DP. Rapid extraction of respiratory waveforms from photoplethysmography: A deep corr-encoder approach. Biomed Signal Process Control. 2023;85:104992. doi:10.1016/j.bspc.2023.104992

**44**. Chin WJ, Chong JW, Cho Y, Cha B, Min SD, Park KS, et al. A novel respiratory rate estimation algorithm from photoplethysmogram using deep learning model. Diagnostics (Basel). 2024;14(3):284. doi:10.3390/diagnostics14030284

**45**. Yang G, Su Q, Li Y, Xu B, Liu H, Li L, et al. Energy-efficient PPG-based respiratory rate estimation using spiking neural networks. Sensors (Basel). 2024;24(12):3980. doi:10.3390/s24123980

**Comment 2.**

*"Supplement technical details including the order of preprocessing filters, β1/β2 parameters of the Adam optimizer, and the division ratio of the model's training/validation sets to improve the reproducibility of the research."*

**Reply:**

We thank the reviewer for these specific suggestions, which directly improve reproducibility. Three technical details have been clarified in the Methods: (i) the Signal preprocessing subsection has been reorganised into in-paragraph subsections with filter type, order, and zero-phase application stated for each stage; (ii) the Adam optimiser configuration now explicitly includes the default momentum parameters (β1 = 0.9, β2 = 0.999), with the optimiser sentence split for clarity; and (iii) the subject-level partition is now stated as five held-out test subjects and 37 (CapnoBase) or 47 (BIDMC) training subjects, with the absence of a separate validation partition noted explicitly.

**Changes made in the manuscript**

**Page 4-5 (Lines 131-151)**

*Signal filtering****.*** The raw PPG is bandpass-filtered between 0.1 and 6.0 Hz using a second-order Butterworth applied bidirectionally for zero phase distortion, which gives an effective fourth-order amplitude response across the cardiac and respiratory bands. The RIAV channel is additionally low-pass filtered at 0.5 Hz and the RIFV channel is bandpass-filtered at 0.1 to 0.6 Hz with the same second-order Butterworth specification.

*Modulation extraction.* Three respiratory modulation channels are derived from the peak-trough series detected on the filtered PPG by adaptive amplitude thresholding: RIIV from the cubic-spline interpolated envelope mean, RIAV from the peak-to-trough amplitude series subsequently low-pass filtered at 0.5 Hz, and RIFV from cubic-spline interpolation of interbeat intervals subsequently bandpass-filtered at 0.1 to 0.6 Hz.

*Segmentation and quality control.* The four channels (filtered PPG, RIIV, RIAV, RIFV) are polyphase-decimated to 30 Hz and segmented into non-overlapping 9.6-second windows of 288 samples. Segments whose mean respiratory rate falls outside the physiological 6 to 36 bpm range are excluded.

*Ground truth preprocessing*. Ground truth signals received separate preprocessing to ensure compatibility with extracted PPG features. Fig 3 illustrates the parallel processing pipelines for capnography (CapnoBase) and impedance pneumography (BIDMC). We evaluated two preprocessing conditions: unfiltered ground truth retaining original frequency content including harmonics, and bandpass-filtered ground truth (0.1 to 0.6 Hz) isolating the primary respiratory band to match the frequency range of extracted PPG features.

*Normalization.* Feature channels and ground-truth waveforms are min-max normalized to [0, 1] per segment, preserving relative temporal dynamics within each segment while ensuring consistent input scales across diverse patients. Respiratory rate values underwent global normalization using dataset-wide bounds (6 to 36 bpm) to maintain consistent regression target scaling across all training samples [47].

**Page 6 (Lines 192-213)**

*Subject-level splitting strategy.* Each dataset was partitioned at the subject level into two non-overlapping subsets. Five subjects from CapnoBase and five subjects from BIDMC were withheld as the held-out evaluation set; the remaining 37 subjects (CapnoBase) and 47 subjects (BIDMC) were used for model training across all five strategies. No subject overlap exists between the training and the held-out evaluation sets within either dataset. Owing to the modest cohort size of both benchmarks, a separate validation set was not extracted; early stopping (patience 15 epochs) and learning-rate reduction on plateau (factor 0.5, patience 7 epochs) monitored the segment-level training loss, while dropout in the encoder (p = 0.1) and in the rate-decoder fully connected layers (p = 0.3 and p = 0.2) provided architectural regularization. Two preprocessing configurations were defined based on ground truth filtering: bandpass-filtered at 0.1 to 0.6 Hz versus unfiltered. Combined with the five training strategies, this yielded 10 experimental conditions.

*Optimizer and training schedule.* All models were trained with the Adam optimizer at an initial learning rate of $10⁻^{3}$ (default momentum parameters $\beta1$ = 0.9, $\beta2$ = 0.999). The global gradient norm was clipped at 1.0 before each parameter update. For the two transfer-learning strategies, the initial learning rate was reduced to $10⁻⁴$ while all other optimizer settings remained identical. An equally weighted Huber loss ($\delta$= 0.1) was applied to both decoder outputs (task weights $\lambda w$ = $\lambda r$ = 1) so that both branches receive balanced gradient signals from the shared encoder. Training ran for a maximum of 100 epochs (batch size 32) with early stopping (patience 15) and learning rate reduction on plateau (factor 0.5, patience 7). Experiments were conducted on a single NVIDIA A100 GPU (40 GB) via Google Colab Pro+.

*Evaluation metrics.* Performance was evaluated using mean absolute error (MAE) and Pearson correlation coefficient (*r*). Waveform MAE quantified pointwise reconstruction error while correlation assessed temporal pattern agreement. For respiratory rate, model outputs were denormalized to breaths per minute before computing MAE. Median values and interquartile ranges across test subjects are reported.

**Comment 3.**

*"Briefly add the application value of the model in specific clinical scenarios (e.g., pediatric anesthesia, ICU monitoring) in the discussion section to strengthen the practical significance of the research."*

**Reply:**

We thank the reviewer for the helpful suggestion. A new Potential Clinical Translation subsection has been added to the Discussion, covering three settings where the framework could be applied: pediatric anesthesia (the CapnoBase context), adult intensive care (the BIDMC context), and early-warning scoring on general wards using NEWS. Each is noted as requiring prospective clinical validation before deployment.

**Changes made in the manuscript**

**Page 11 (Lines 387-396)**

**Potential Clinical Translation**

We see three clinical contexts where this framework could be useful, though prospective validation is needed before deployment in any of them. During pediatric anesthesia, from which the CapnoBase recordings originate [16, 51], capnography is standard but often interrupted at mask induction and emergence; a PPG sensor already in place for pulse oximetry could provide continuous respiratory estimates at precisely these moments. In adult intensive care, represented by the BIDMC patients [32, 52], movement and electrode repositioning often disrupt impedance pneumography traces, and the same PPG signal could fill these monitoring gaps. On general wards, respiratory rate remains the vital sign least reliably recorded in routine nursing rounds [4], and a wearable PPG device running this framework could deliver the continuous respiratory data that NEWS scoring [3] needs but rarely receives.

**References**

**3.** Bilben B, Grandal L, Søvik S. National Early Warning Score (NEWS) as an emergency department predictor of disease severity and 90-day survival in the acutely dyspneic patient – a prospective observational study. Scand J Trauma Resusc Emerg Med. 2016;24(1):80. doi:10.1186/s13049-016-0273-9

**4.** L'Her E, N'Guyen QT, Pateau V, Bodenes L, Lellouche F. Photoplethysmographic determination of the respiratory rate in acutely ill patients: Validation of a new algorithm and implementation into a biomedical device. Ann Intensive Care. 2019;9(1):11. doi:10.1186/s13613-019-0485-z

**16.** Karlen W, Raman S, Ansermino JM, Dumont GA. Multiparameter respiratory rate estimation from the photoplethysmogram. IEEE Trans Biomed Eng. 2013;60(7):1946–53. doi:10.1109/TBME.2013.2246160

**32.** Pimentel MAF, Charlton PH, Clifton DA. Toward a robust estimation of respiratory rate from pulse oximeters. IEEE Trans Biomed Eng. 2017;64(8):1914–23. doi:10.1109/TBME.2016.2613124

**51**. Karlen W, Turner M, Cooke E, Dumont GA, Ansermino JM. CapnoBase: Signal database and tools to collect, share and annotate respiratory signals. In: 2010 Annual Meeting of the Society for Technology in Anesthesia. Society for Technology in Anesthesia; 2010. p. 25.

**52.** Goldberger AL, Amaral LAN, Glass L, Hausdorff JM, Ivanov PC, Mark RG, et al. PhysioBank, PhysioToolkit, and PhysioNet: Components of a new research resource for complex physiologic signals. Circulation. 2000;101(23):e215–20. doi:10.1161/01.CIR.101.23.e215

**Reviewer 3**

**Comment.**

*"However, the occurrence of anti-phase waveform reconstructions in 'worst-case' segments and the regression-to-the-mean effect at higher respiratory rates suggest opportunities for further refinement of the loss functions or polarity correction."*

**Reply:**

We thank the reviewer for the constructive observation. The anti-phase waveform reconstructions are discussed in the Limitations and Future Work subsection, where we note that a pointwise loss cannot capture temporal alignment and suggest that refinements of the loss function or polarity correction may improve reconstruction consistency. The regression-toward-the-mean effect reflects the narrow rate distribution in the BIDMC training data, which we acknowledge as an open limitation; rate-balanced sampling is one direction for future investigation.

**Changes made in the manuscript**

**Page 13 (Lines 443-446)**

We observed phase-inverted waveform reconstructions in a minority of segments, where predicted peaks aligned with ground truth troughs despite the dominant respiratory frequency being preserved; this may reflect the inability of a pointwise loss to capture temporal alignment, suggesting that refinements of the loss function or polarity correction may improve reconstruction consistency.
