# Supplementary material for: A late fusion multi-task learning for respiratory waveform and rate estimation from photoplethysmography
Source: PLoS One. 2026 Jul 10;21(7):e0353203. doi: 10.1371/journal.pone.0353203 (PMC13353955; doi:10.1371/journal.pone.0353203)
Supplement: Peer review history

# Peer review: Decision Letter 0

**DOI:** 10.1371/journal.pone.0353203.r001

**Authors:** Haipeng Liu

21 Apr 2026

PONE-D-26-10284

A late fusion multi-task learning for respiratory waveform and rate estimation from photoplethysmography

PLOS One

Dear Dr. Nguyen,

Thank you for submitting your manuscript to PLOS ONE. After careful consideration, we feel that it has merit but does not fully meet PLOS ONE’s publication criteria as it currently stands. Therefore, we invite you to submit a revised version of the manuscript that addresses the points raised during the review process.

Please submit your revised manuscript by Jun 05 2026 11:59PM. If you will need more time than this to complete your revisions, please reply to this message or contact the journal office at plosone@plos.org. When you're ready to submit your revision, log on to https://www.editorialmanager.com/pone/ and select the 'Submissions Needing Revision' folder to locate your manuscript file.

Please include the following items when submitting your revised manuscript:

- A letter that responds to each point raised by the academic editor and reviewer(s). You should upload this letter as a separate file labeled 'Response to Reviewers'.
- A marked-up copy of your manuscript that highlights changes made to the original version. You should upload this as a separate file labeled 'Revised Manuscript with Track Changes'.
- An unmarked version of your revised paper without tracked changes. You should upload this as a separate file labeled 'Manuscript'.

If you would like to make changes to your financial disclosure, please include your updated statement in your cover letter. Guidelines for resubmitting your figure files are available below the reviewer comments at the end of this letter.

If applicable, we recommend that you deposit your laboratory protocols in protocols.io to enhance the reproducibility of your results. Protocols.io assigns your protocol its own identifier (DOI) so that it can be cited independently in the future. For instructions see: https://journals.plos.org/plosone/s/submission-guidelines#loc-laboratory-protocols. Additionally, PLOS ONE offers an option for publishing peer-reviewed Lab Protocol articles, which describe protocols hosted on protocols.io. Read more information on sharing protocols at https://plos.org/protocols?utm_medium=editorial-email&utm_source=authorletters&utm_campaign=protocols.

As the corresponding author, your ORCID iD is verified in the submission system and will appear in the published article. PLOS supports the use of ORCID, and we encourage all coauthors to register for an ORCID iD and use it as well. Please encourage your coauthors to verify their ORCID iD within the submission system before final acceptance, as unverified ORCID iDs will not appear in the published article. *Only*  the individual author can complete the verification step; PLOS staff *cannot*  verify ORCID iDs on behalf of authors.

We look forward to receiving your revised manuscript.

Kind regards,

Haipeng Liu

Academic Editor

PLOS One

Journal Requirements:

When submitting your revision, we need you to address these additional requirements.

1. Please ensure that your manuscript meets PLOS ONE's style requirements, including those for file naming. The PLOS ONE style templates can be found at

https://journals.plos.org/plosone/s/file?id=wjVg/PLOSOne_formatting_sample_main_body.pdf and

https://journals.plos.org/plosone/s/file?id=ba62/PLOSOne_formatting_sample_title_authors_affiliations.pdf

2. Please note that PLOS One has specific guidelines on code sharing for submissions in which author-generated code underpins the findings in the manuscript. In these cases, we expect all author-generated code to be made available without restrictions upon publication of the work. Please review our guidelines at https://journals.plos.org/plosone/s/materials-and-software-sharing#loc-sharing-code and ensure that your code is shared in a way that follows best practice and facilitates reproducibility and reuse.

3. Please update your submission to use the PLOS LaTeX template. The template and more information on our requirements for LaTeX submissions can be found at http://journals.plos.org/plosone/s/latex.

4. Thank you for uploading your study's underlying data set. Unfortunately, the repository you have noted in your Data Availability statement does not qualify as an acceptable data repository according to PLOS's standards.

At this time, please upload the minimal data set necessary to replicate your study's findings to a stable, public repository (such as figshare or Dryad) and provide us with the relevant URLs, DOIs, or accession numbers that may be used to access these data. For a list of recommended repositories and additional information on PLOS standards for data deposition, please see https://journals.plos.org/plosone/s/recommended-repositories.

5. If the reviewer comments include a recommendation to cite specific previously published works, please review and evaluate these publications to determine whether they are relevant and should be cited. There is no requirement to cite these works unless the editor has indicated otherwise.

[Note: HTML markup is below. Please do not edit.]

Reviewers' comments:

Reviewer's Responses to Questions

**Comments to the Author**

1. Is the manuscript technically sound, and do the data support the conclusions?

The manuscript must describe a technically sound piece of scientific research with data that supports the conclusions. Experiments must have been conducted rigorously, with appropriate controls, replication, and sample sizes. The conclusions must be drawn appropriately based on the data presented.

Reviewer #1: Yes

Reviewer #2: Yes

Reviewer #3: Yes

**********

2. Has the statistical analysis been performed appropriately and rigorously?

Reviewer #1: Yes

Reviewer #2: Yes

Reviewer #3: Yes

**********

3. Have the authors made all data underlying the findings in their manuscript fully available?

The PLOS Data policy requires authors to make all data underlying the findings described in their manuscript fully available without restriction, with rare exception (please refer to the Data Availability Statement in the manuscript PDF file). The data should be provided as part of the manuscript or its supporting information, or deposited to a public repository. For example, in addition to summary statistics, the data points behind means, medians and variance measures should be available. If there are restrictions on publicly sharing data—e.g. participant privacy or use of data from a third party—those must be specified.

Reviewer #1: Yes

Reviewer #2: Yes

Reviewer #3: Yes

**********

4. Is the manuscript presented in an intelligible fashion and written in standard English?

PLOS ONE does not copyedit accepted manuscripts, so the language in submitted articles must be clear, correct, and unambiguous. Any typographical or grammatical errors should be corrected at revision, so please note any specific errors here.

Reviewer #1: Yes

Reviewer #2: Yes

Reviewer #3: Yes

**********

5. Review Comments to the Author

Please use the space provided to explain your answers to the questions above. You may also include additional comments for the author, including concerns about dual publication, research ethics, or publication ethics. (Please upload your review as an attachment if it exceeds 20,000 characters)

Reviewer #1: General comments:

Excellent scientific foundation, clear articulation of the research gap and contribution, methodology is extremely strong, architecture description is excellent, and the results deliver insightful interpretations.

Minor recommendations:

Strengthen the abstract to give a clear summary, strong contribution, and quantitative results to emphasize relevance and key results only. Same goes for the introduction section, I would recommend to streamline and reduce redundancy, and move the physiological detail to Methods.

For the Results chapter I would recommend to summarize key findings first, then elaborate. In the Discussion section limitations and future work could be included. Repeating metrics already presented could be avoided.

Conclusion:

Scientifically very strong with minor recommendations.

Reviewer #2: This paper proposes a physiology-based late fusion multi-task learning framework for the joint estimation of respiratory waveforms and respiratory rates from PPG signals. The research topic aligns with the clinical demands for non-invasive respiratory monitoring, featuring a scientific research design, sufficient experimental validation, and detailed result analysis. Methodologically, the framework integrates RIIV/RIAV/RIFV physiological modulation features with deep learning, achieving both innovation and interpretability; its systematic analysis of training strategies and reference signal filtering provides valuable insights for related studies. The paper is well-structured in writing and represents a solid and impactful research work. Minor Revision is recommended.

1. It is suggested to add a concise comparison table in the "Comparison with existing methods" section, which summarizes the optimal results of this study and the core metrics (e.g., respiratory rate MAE, waveform MAE/correlation) of recent representative studies such as Davies and Mandic (2023) and Chin et al. (2024) on the CapnoBase and BIDMC datasets, so as to intuitively reflect the performance positioning.

2. Supplement technical details including the order of preprocessing filters, β1/β2 parameters of the Adam optimizer, and the division ratio of the model's training/validation sets to improve the reproducibility of the research.

3. Briefly add the application value of the model in specific clinical scenarios (e.g., pediatric anesthesia, ICU monitoring) in the discussion section to strengthen the practical significance of the research.

Reviewer #3: This study presents a robust late-fusion multitask framework that effectively leverages physiologically grounded RIIV, RIAV, and RIFV modulations to estimate respiratory waveforms and rates simultaneously. A standout contribution is the demonstration that sequential transfer learning significantly outperforms combined training by mitigating the morphological conflicts between capnography and impedance pneumography reference signals. The model's ability to provide accurate rate estimates from 9.6-second windows, achieving mean absolute errors (MAEs) of 1.33 bpm on BIDMC and 2.27 bpm on CapnoBase, offers substantial clinical value for real-time monitoring. However, the occurrence of anti-phase waveform reconstructions in "worst-case" segments and the regression-to-the-mean effect at higher respiratory rates suggest opportunities for further refinement of the loss functions or polarity correction.

**********

6. PLOS authors have the option to publish the peer review history of their article (what does this mean?). If published, this will include your full peer review and any attached files.

If you choose “no”, your identity will remain anonymous but your review may still be made public.

**Do you want your identity to be public for this peer review?**  For information about this choice, including consent withdrawal, please see our Privacy Policy.

Reviewer #1: **Yes:**  Vera Hartmann

Reviewer #2: No

Reviewer #3: **Yes:**  Adan Khan

**********

[NOTE: If reviewer comments were submitted as an attachment file, they will be attached to this email and accessible via the submission site. Please log into your account, locate the manuscript record, and check for the action link "View Attachments". If this link does not appear, there are no attachment files.]

To ensure your figures meet our technical requirements, please review our figure guidelines: https://journals.plos.org/plosone/s/figures

You may also use PLOS’s free figure tool, NAAS, to help you prepare publication quality figures: https://journals.plos.org/plosone/s/figures#loc-tools-for-figure-preparation.

NAAS will assess whether your figures meet our technical requirements by comparing each figure against our figure specifications.

# Author comment: Author response to Decision Letter 1

**DOI:** 10.1371/journal.pone.0353203.r002

2 Jun 2026

Please see the attached 'Response to Reviewers' document for our detailed point-by-point responses to all reviewer and editor comments.

Attachment Submitted filename: Response_to_Reviewers.docx

# Peer review: Decision Letter 1

**DOI:** 10.1371/journal.pone.0353203.r003

**Authors:** Haipeng Liu

21 Jun 2026

A late fusion multi-task learning for respiratory waveform and rate estimation from photoplethysmography

PONE-D-26-10284R1

Dear Dr. Nguyen,

We’re pleased to inform you that your manuscript has been judged scientifically suitable for publication and will be formally accepted for publication once it meets all outstanding technical requirements.

Within one week, you’ll receive an e-mail detailing the required amendments. When these have been addressed, you’ll receive a formal acceptance letter and your manuscript will be scheduled for publication.

An invoice will be generated when your article is formally accepted. Please note, if your institution has a publishing partnership with PLOS and your article meets the relevant criteria, all or part of your publication costs will be covered. Please make sure your user information is up-to-date by logging into Editorial Manager at Editorial Manager® and clicking the ‘Update My Information' link at the top of the page. For questions related to billing, please contact billing support.

If your institution or institutions have a press office, please notify them about your upcoming paper to help maximize its impact. If they’ll be preparing press materials, please inform our press team as soon as possible -- no later than 48 hours after receiving the formal acceptance. Your manuscript will remain under strict press embargo until 2 pm Eastern Time on the date of publication. For more information, please contact onepress@plos.org.

Kind regards,

Haipeng Liu

Academic Editor

PLOS One

Additional Editor Comments (optional):

Reviewers' comments:

Reviewer's Responses to Questions

**Comments to the Author**

1. If the authors have adequately addressed your comments raised in a previous round of review and you feel that this manuscript is now acceptable for publication, you may indicate that here to bypass the “Comments to the Author” section, enter your conflict of interest statement in the “Confidential to Editor” section, and submit your "Accept" recommendation.

Reviewer #1: All comments have been addressed

Reviewer #2: All comments have been addressed

**********

2. Is the manuscript technically sound, and do the data support the conclusions?

The manuscript must describe a technically sound piece of scientific research with data that supports the conclusions. Experiments must have been conducted rigorously, with appropriate controls, replication, and sample sizes. The conclusions must be drawn appropriately based on the data presented.

Reviewer #1: Yes

Reviewer #2: Yes

**********

3. Has the statistical analysis been performed appropriately and rigorously?

Reviewer #1: Yes

Reviewer #2: Yes

**********

4. Have the authors made all data underlying the findings in their manuscript fully available?

The PLOS Data policy requires authors to make all data underlying the findings described in their manuscript fully available without restriction, with rare exception (please refer to the Data Availability Statement in the manuscript PDF file). The data should be provided as part of the manuscript or its supporting information, or deposited to a public repository. For example, in addition to summary statistics, the data points behind means, medians and variance measures should be available. If there are restrictions on publicly sharing data—e.g. participant privacy or use of data from a third party—those must be specified.

Reviewer #1: Yes

Reviewer #2: Yes

**********

5. Is the manuscript presented in an intelligible fashion and written in standard English?

PLOS ONE does not copyedit accepted manuscripts, so the language in submitted articles must be clear, correct, and unambiguous. Any typographical or grammatical errors should be corrected at revision, so please note any specific errors here.

Reviewer #1: Yes

Reviewer #2: Yes

**********

6. Review Comments to the Author

Please use the space provided to explain your answers to the questions above. You may also include additional comments for the author, including concerns about dual publication, research ethics, or publication ethics. (Please upload your review as an attachment if it exceeds 20,000 characters)

Reviewer #1: The manuscript presents a technically sound study with a clearly defined methodological pipeline, including preprocessing, model architecture, and evaluation strategy. The experimental design, especially the comparison of five training strategies and two preprocessing conditions is appropriate and supports the conclusions. The results are interpreted cautiously, and limitations (e.g., waveform inversion, dataset constraints) are transparently discussed.

The statistical evaluation using MAE and Pearson correlation is appropriate for the study objectives. Reporting of median and interquartile ranges across subjects is suitable given the dataset size. Minor improvements could include explicitly justifying the choice of metrics (e.g., why MAE over RMSE) and potentially adding statistical significance testing when comparing training strategies.

Overall, the manuscript is now technically sound, well-written, and provides a meaningful contribution to the field of physiological signal analysis and AI-based respiratory monitoring. I recommend acceptance without improvements.

Reviewer #2: I have carefully reviewed the author's revised manuscript (PONE-D-26-10284_R1) and their responses. The author has completely and accurately addressed all the core revision suggestions I raised in the previous round. The revised manuscript demonstrates significant improvement in the clarity of result comparisons, the reproducibility of the research, and the elaboration of its clinical significance, and it has met the publication standards.

Confirmation of Revisions:

1.Quantitative Result Comparison: The author has added Table 2 in the "Discussion" section. This table presents the optimal results of this study alongside key metrics from recent works such as Davies and Mandic (2023) and Chin et al. (2024) on the same datasets (CapnoBase, BIDMC), and it notes the differences in evaluation protocols. This fully satisfies the requirement.

2.Supplementation of Technical Details: The author has clearly supplemented the following in the "Materials and Methods" section:

All filters are second-order Butterworth filters. The Adam optimizer parameters are β1=0.9, β2=0.999.

A detailed description of the dataset partitioning strategy: "Each dataset was partitioned at the subject level... Five subjects from CapnoBase and five subjects from BIDMC were withheld as the held-out evaluation set; the remaining 37 subjects (CapnoBase) and 47 subjects (BIDMC) were used for model training...". Furthermore, it explains that due to the limited cohort sizes, a separate validation set was not created. Instead, strategies such as "early stopping" and "learning-rate reduction on plateau" were employed for model selection and regularization.

3.Strengthening of Clinical Significance: The author has added a new subsection titled "Potential Clinical Translation" in the "Discussion" section, which explicitly elaborates on the model's application potential in specific scenarios such as pediatric anesthesia, ICU monitoring, and general ward settings.

Recommendation:

Accept

**********

7. PLOS authors have the option to publish the peer review history of their article (what does this mean?). If published, this will include your full peer review and any attached files.

If you choose “no”, your identity will remain anonymous but your review may still be made public.

**Do you want your identity to be public for this peer review?**  For information about this choice, including consent withdrawal, please see our Privacy Policy.

Reviewer #1: **Yes:**  Vera Hartmann

Reviewer #2: No

**********

# Editor report: Acceptance letter

**DOI:** 10.1371/journal.pone.0353203.r004

**Authors:** Haipeng Liu

PONE-D-26-10284R1

PLOS One

Dear Dr. Nguyen,

I'm pleased to inform you that your manuscript has been deemed suitable for publication in PLOS One. Congratulations! Your manuscript is now being handed over to our production team.

At this stage, our production department will prepare your paper for publication. This includes ensuring the following:

* All references, tables, and figures are properly cited

* All relevant supporting information is included in the manuscript submission,

* There are no issues that prevent the paper from being properly typeset

You will receive further instructions from the production team, including instructions on how to review your proof when it is ready. Please keep in mind that we are working through a large volume of accepted articles, so please give us a few days to review your paper and let you know the next and final steps.

Lastly, if your institution or institutions have a press office, please let them know about your upcoming paper now to help maximize its impact. If they'll be preparing press materials, please inform our press team within the next 48 hours. Your manuscript will remain under strict press embargo until 2 pm Eastern Time on the date of publication. For more information, please contact onepress@plos.org.

You will receive an invoice from PLOS for your publication fee after your manuscript has reached the completed accept phase. If you receive an email requesting payment before acceptance or for any other service, this may be a phishing scheme. Learn how to identify phishing emails and protect your accounts at https://explore.plos.org/phishing.

If we can help with anything else, please email us at customercare@plos.org.

Thank you for submitting your work to PLOS ONE and supporting open access.

Kind regards,

PLOS ONE Editorial Office Staff

on behalf of

Dr. Haipeng Liu

Academic Editor

PLOS One
